# Supplementary figures and images for: Development and external validation of a multivariate model for predicting pneumonia in patients receiving maintenance hemodialysis: a retrospective study
Source: PeerJ. 2025 Oct 9;13:e20070. doi: 10.7717/peerj.20070 (PMC12515429; doi:10.7717/peerj.20070)

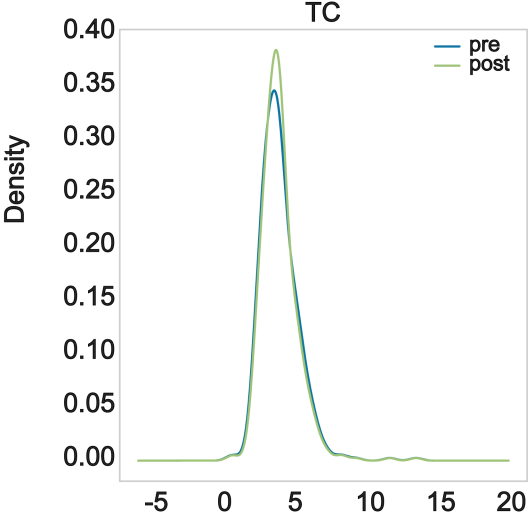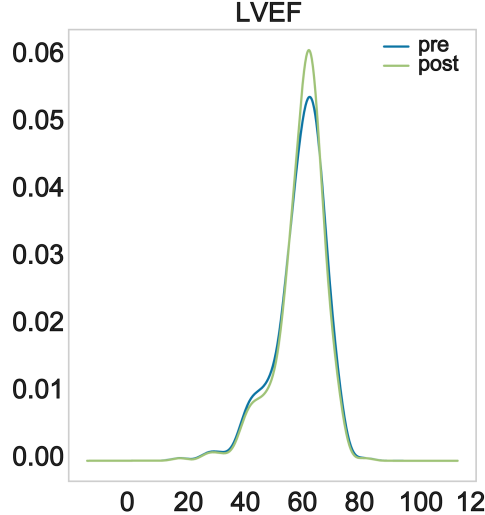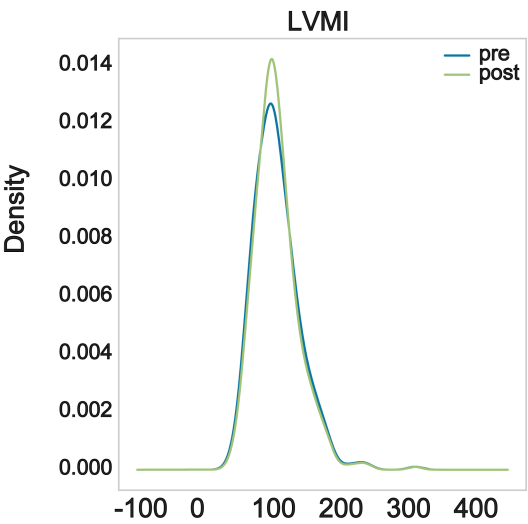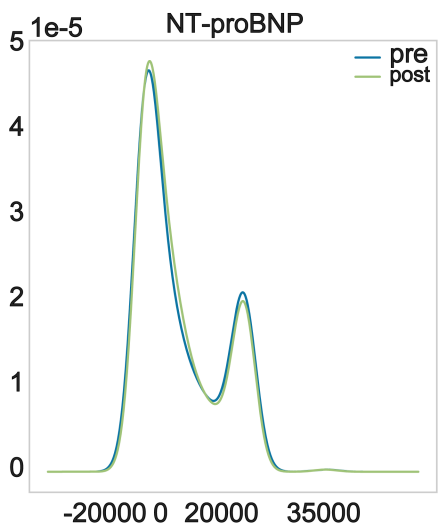

Supplement: Supplemental Information 3 [file peerj-13-20070-s003.pdf]

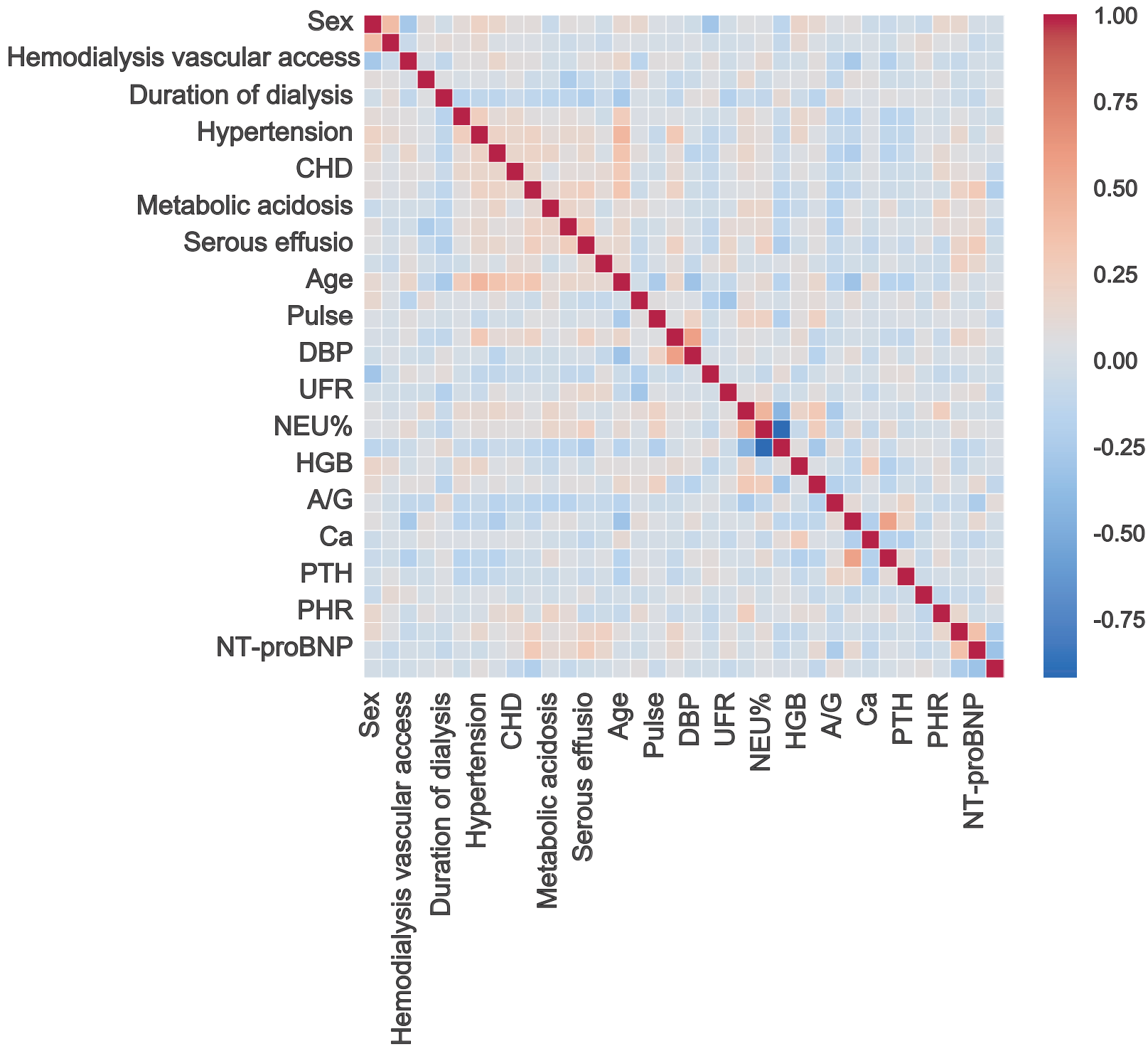

Supplement: Supplemental Information 4 [file peerj-13-20070-s004.pdf]

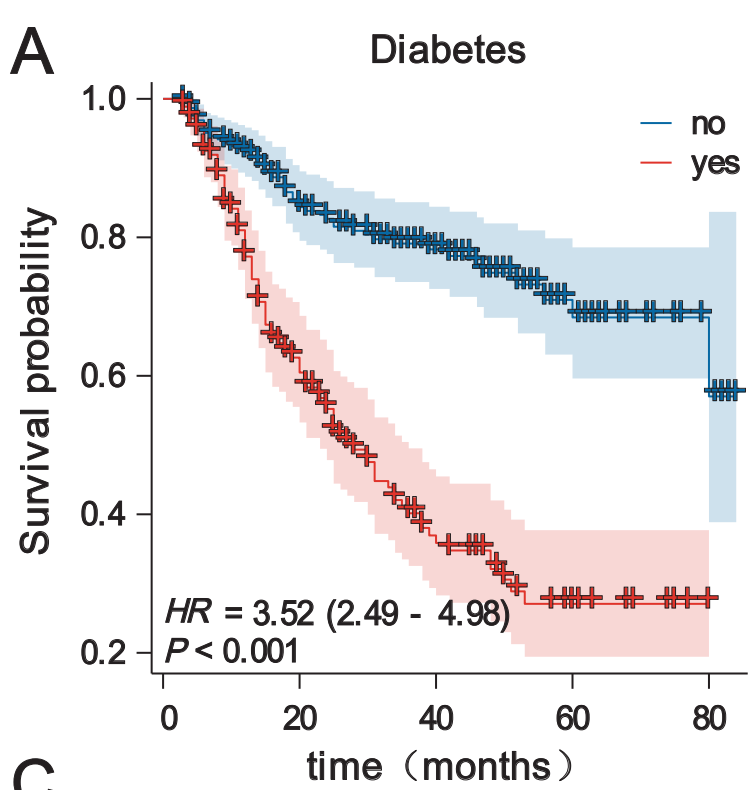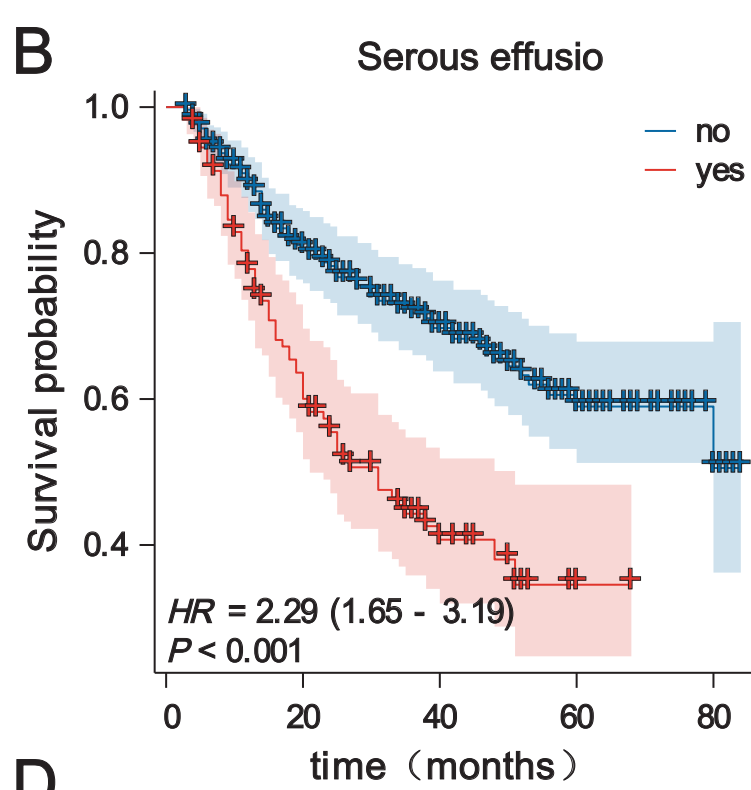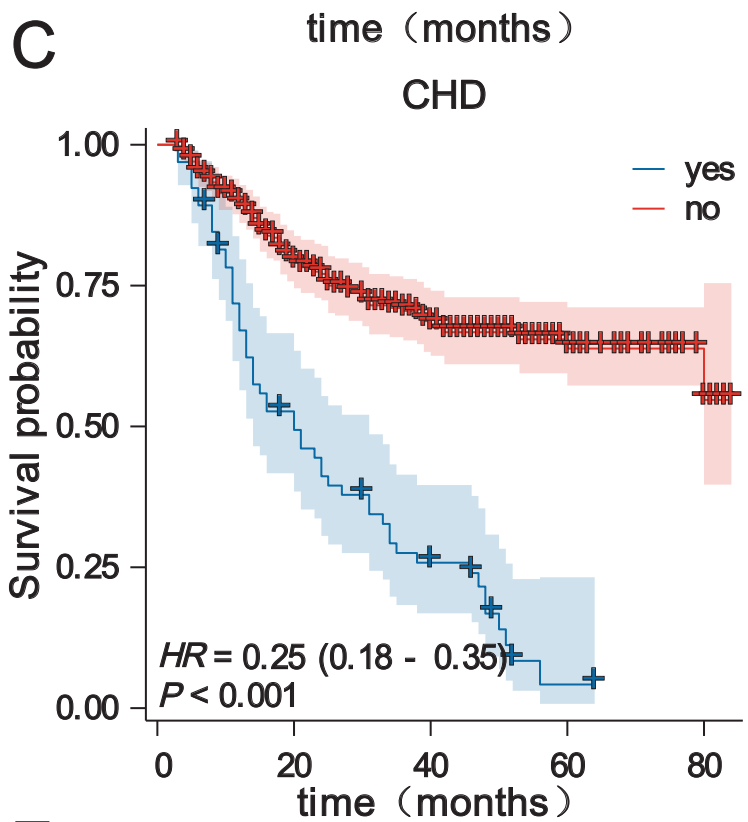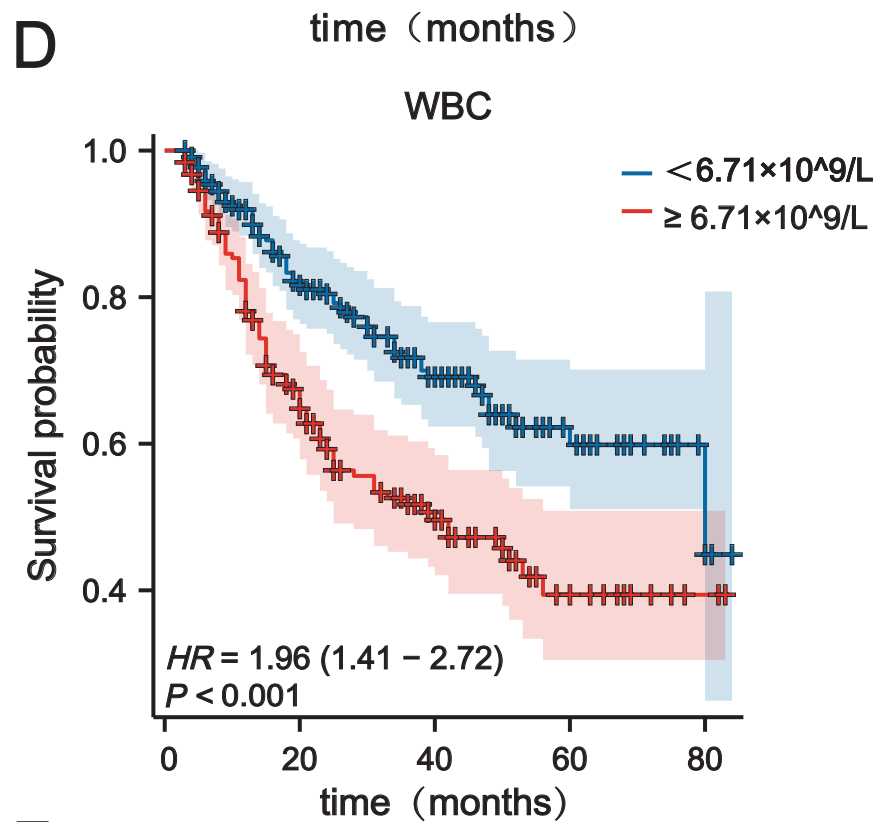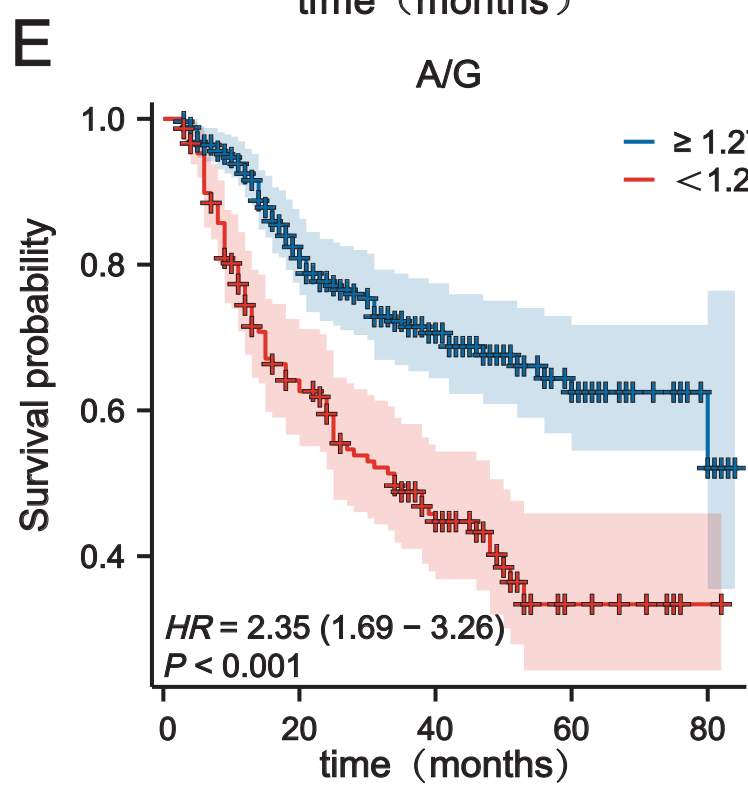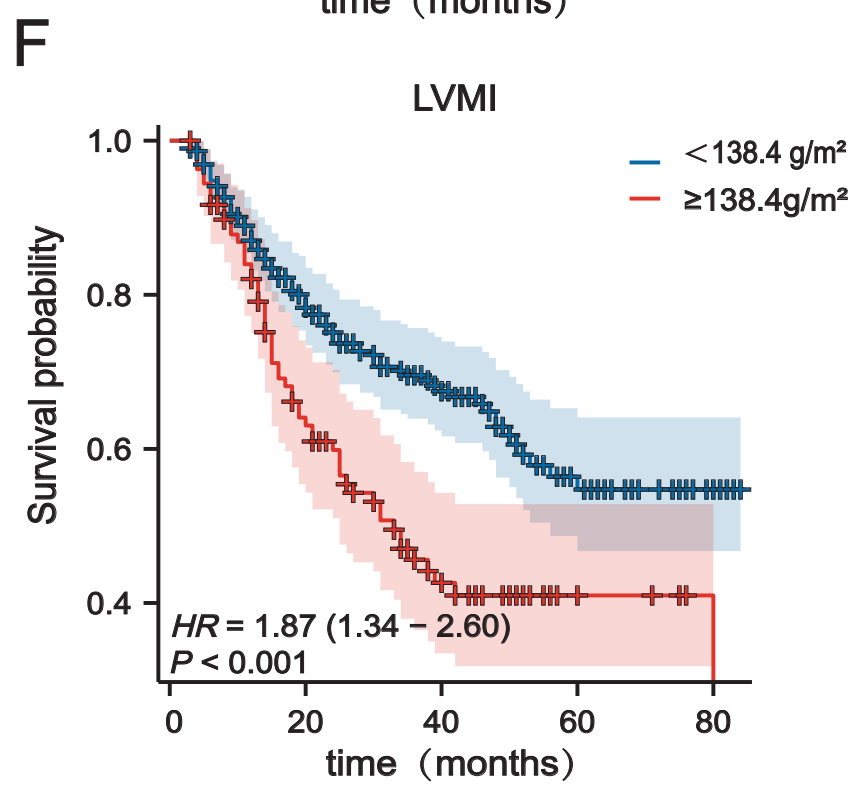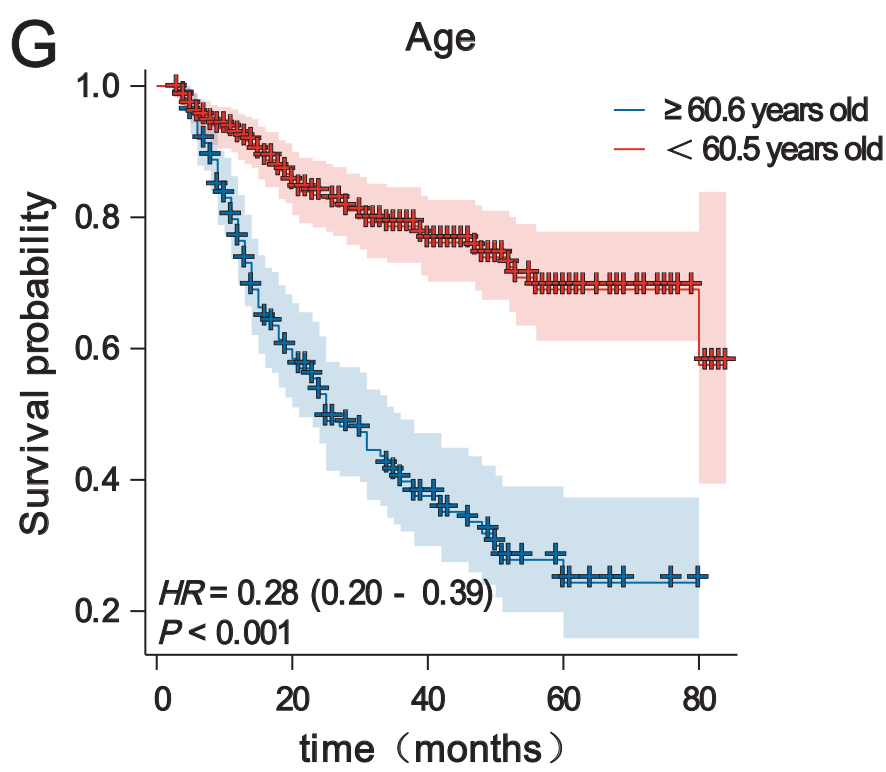

Supplement: Supplemental Information 6 [file peerj-13-20070-s006.pdf]
