# Supplementary material for: Development and external validation of a multivariate model for predicting pneumonia in patients receiving maintenance hemodialysis: a retrospective study
Source: PeerJ. 2025 Oct 9;13:e20070. doi: 10.7717/peerj.20070 (PMC12515429; doi:10.7717/peerj.20070)
Supplement: Supplemental Information 5 [file peerj-13-20070-s005.pdf]

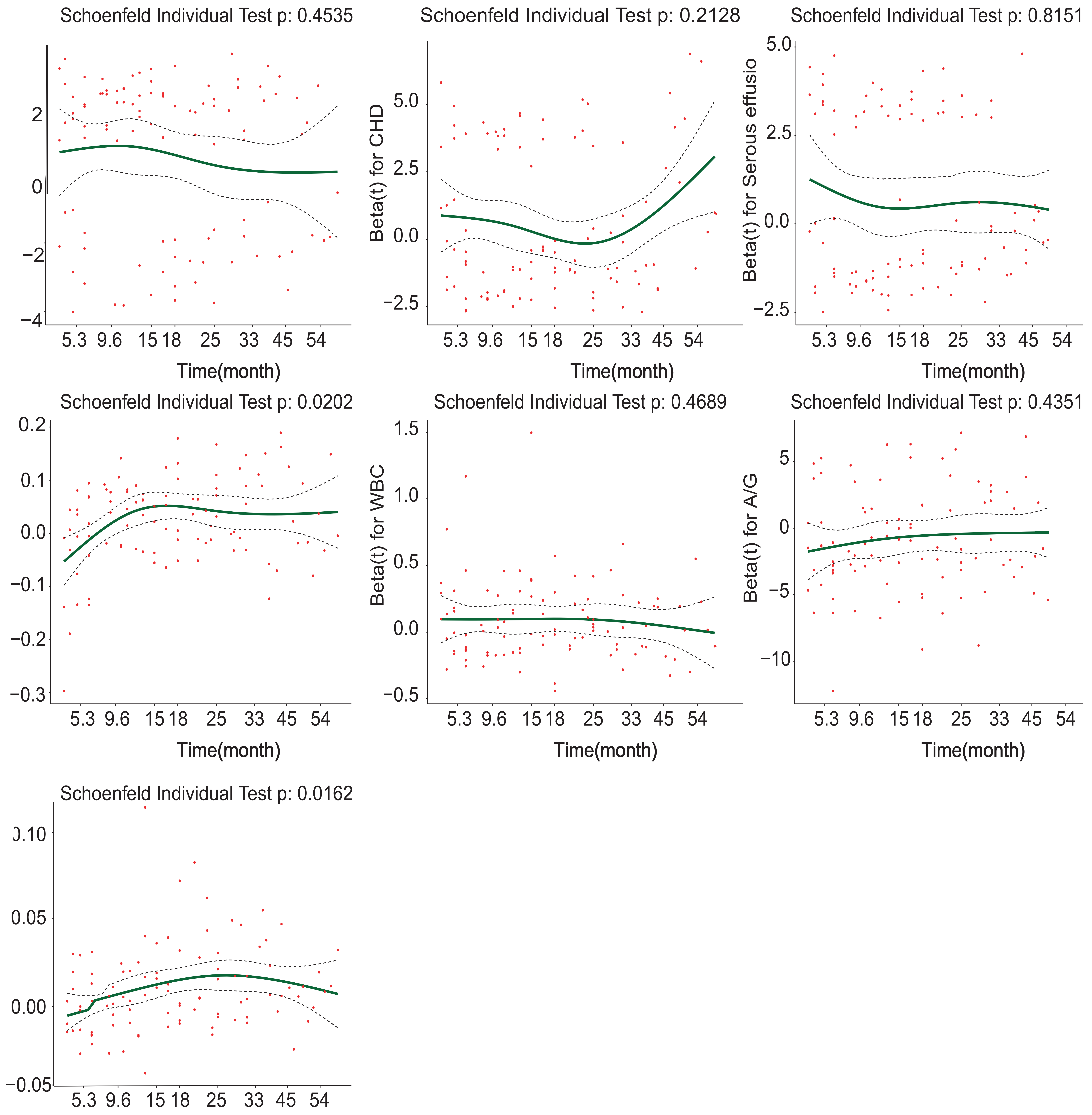

Schoenfeld Individual Test p: 0.4535
2
0
−2
−4
5.3 9.6 15 18 25 33 45 54
Time(month)
Schoenfeld Individual Test p: 0.2128
Beta(t) for CHD
5.0
2.5
0.0
−2.5
5.3 9.6 15 18 25 33 45 54
Time(month)
Schoenfeld Individual Test p: 0.8151
Beta(t) for Serous effusio
5.0
2.5
0.0
−2.5
5.3 9.6 15 18 25 33 45 54
Time(month)
Schoenfeld Individual Test p: 0.0202
0.2
0.1
0.0
−0.1
−0.2
−0.3
5.3 9.6 15 18 25 33 45 54
Time(month)
Schoenfeld Individual Test p: 0.4689
Beta(t) for WBC
1.5
1.0
0.5
0.0
−0.5
5.3 9.6 15 18 25 33 45 54
Time(month)
Schoenfeld Individual Test p: 0.4351
Beta(t) for A/G
5
0
−5
−10
5.3 9.6 15 18 25 33 45 54
Time(month)
Schoenfeld Individual Test p: 0.0162
0.05
0.00
−0.05
5.3 9.6 15 18 25 33 45 54
