# Supplementary material for: Development and external validation of a multivariate model for predicting pneumonia in patients receiving maintenance hemodialysis: a retrospective study
Source: PeerJ. 2025 Oct 9;13:e20070. doi: 10.7717/peerj.20070 (PMC12515429; doi:10.7717/peerj.20070)
Supplement: Supplemental Information 7 [file peerj-13-20070-s007.docx]

| **Table S1. Abbreviations** | |
| --- | --- |
| A/G | albumin-globulin ratio |
| BMI | body mass index |
| Ca | serum calcium |
| CHD | coronary heart disease |
| CKD | chronic kidney disease |
| CRP | C-reactive protein |
| DCA | decision curve analysis |
| HF | heart failure |
| HGB | hemoglobin |
| IQR | interquartile range |
| K-M | Kaplan-Meier |
| Lasso | Least Absolute Shrinkage and Selection Operator |
| LVEF | Echocardiographic data: left ventricular ejection fraction |
| LVMI | left ventricular mass index |
| LYM% | lymphocyte ratio |
| MHD | maintenance hemodialysis |
| NEU% | neutrophil ratio |
| NT-proBNP | N-terminal prohormone of brain natriuretic peptide |
| NYHA | New York Heart Association |
| P | serum phosphorus |
| PHR | Platelet to high-density lipoprotein cholesterol ratio |
| PTH | Parathyroid hormone |
| Scr | Serum creatinine |
| T1DM | type 1 diabetes mellitus |
| T2DM | type 2 diabetes mellitus |
| TC | Total Cholesterol |
| UFR | Ultrafiltration Rate |
| URR | urea reduction ratio |
| WBC | white blood cell |
