# Supplementary material for: Development and external validation of a multivariate model for predicting pneumonia in patients receiving maintenance hemodialysis: a retrospective study
Source: PeerJ. 2025 Oct 9;13:e20070. doi: 10.7717/peerj.20070 (PMC12515429; doi:10.7717/peerj.20070)
Supplement: Supplemental Information 8 [file peerj-13-20070-s008.docx]

| **Table S2 & Figure S1** | | | | | | | | |
| --- | --- | --- | --- | --- | --- | --- | --- | --- |
| Table S2A Variable description before multiple interpolation of data | | | | | | | | |
| ***variable*** | ***total*** | ***mean value*** | ***median*** | ***25 % quantiles*** | ***75 % quantiles*** | ***minimum value*** | ***maximum value*** | ***Defect rate %*** |
| TC | 362 | 4.209 | 4.025 | 3.332 | 4.842 | 0.75 | 13.86 | 10.617 |
| LVEF | 361 | 62.242 | 64 | 58 | 68 | 20 | 85 | 10.864 |
| LVMI | 362 | 124.131 | 118.404 | 99.929 | 141.73 | 54.714 | 329.034 | 10.617 |
| NT-proBNP | 366 | 13230.021 | 7214 | 2049.09 | 23919 | 104.7 | 62403.76 | 9.63 |
| Table S2B Variable description after multiple interpolation of data | | | | | | | | |
| ***variable*** | ***total*** | ***mean value*** | ***median*** | ***25 % quantiles*** | ***75 % quantiles*** | ***minimum value*** | ***maximum value*** |  |
| TC | 405 | 4.198 | 4.03 | 3.4 | 4.72 | 0.75 | 13.86 |  |
| LVEF | 405 | 62.42 | 64 | 59 | 68 | 20 | 85 |  |
| LVMI | 405 | 123.817 | 118.556 | 102.028 | 139.631 | 54.714 | 329.034 |  |
| NT-proBNP | 405 | 12909.823 | 7680.01 | 2255 | 22594 | 104.7 | 62403.76 |  |
| Figure S1. Data density distribution map | | | | | | | | |
| 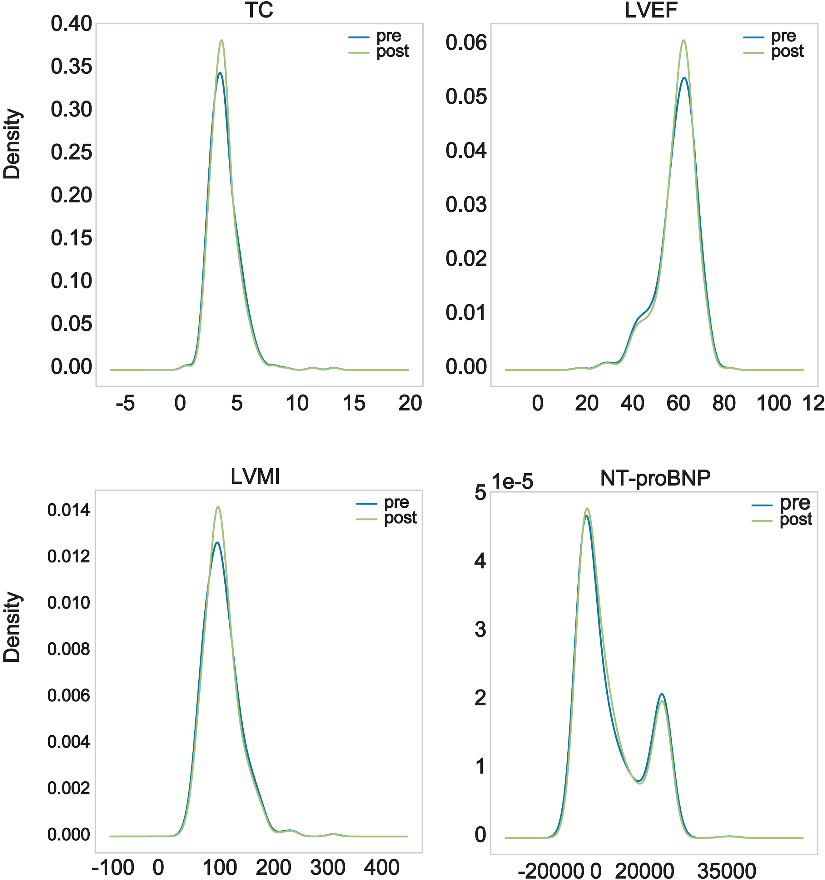 | | | | | | | | |
| **Note:** TC, Total Cholesterol; LVEF, left ventricular ejection fraction；LVMI, left atrial diameter; NT-proBNP, N-terminal prohormone of brain natriuretic peptide. | | | | | | | | |
