# Supplementary material for: Development and external validation of a multivariate model for predicting pneumonia in patients receiving maintenance hemodialysis: a retrospective study
Source: PeerJ. 2025 Oct 9;13:e20070. doi: 10.7717/peerj.20070 (PMC12515429; doi:10.7717/peerj.20070)
Supplement: Supplemental Information 9 [file peerj-13-20070-s009.docx]

| **Table S3. Single-factor Cox analysis** | | | | |
| --- | --- | --- | --- | --- |
| **Variables** | ***n*** | ***HR*** | **95%*CI*** | ***P-value*** |
| Age(years) | 290 | 1.044 | [1.030,1.058] | ＜0.001 |
| BMI(kg/m²) | 290 | 1.009 | [0.957,1.063] | 0.752 |
| Pulse(bpm) | 290 | 1.012 | [0.998,1.027] | 0.094 |
| SBP(mmHg) | 290 | 1.006 | [0.999,1.014] | 0.096 |
| DBP(mmHg) | 290 | 0.993 | [0.980,1.006] | 0.288 |
| URR(%) | 290 | 0.993 | [0.968,1.020] | 0.615 |
| UFR(ml/kg*h) | 290 | 1.02 | [0.974,1.068] | 0.406 |
| WBC(10^9/L) | 290 | 1.136 | [1.076,1.199] | ＜0.001 |
| NEU% | 290 | 1.036 | [1.016,1.056] | ＜0.001 |
| LYM% | 290 | 0.951 | [0.926,0.976] | ＜0.001 |
| HGB(g/L) | 290 | 1.001 | [0.991,1.011] | 0.864 |
| CRP | 290 | 1.005 | [1.002,1.009] | 0.002 |
| A/G | 290 | 0.185 | [0.093,0.365] | ＜0.001 |
| Scr(μmol/L) | 290 | 0.999 | [0.999,1.000] | 0.065 |
| Ca(mmol/L) | 290 | 1.472 | [0.752,2.882] | 0.259 |
| P(mmol/L) | 290 | 0.732 | [0.538,0.995] | 0.046 |
| PTH(pmol/L) | 290 | 0.998 | [0.997,0.999] | 0.003 |
| TC(mmol/L) | 290 | 0.88 | [0.741,1.045] | 0.144 |
| PHR(10^9 mmol/L^2) | 290 | 1.001 | [1.000,1.002] | 0.07 |
| LVMI (g/m²) | 290 | 1.01 | [1.005,1.015] | ＜0.001 |
| ln（NT-proBNP）(pg/mL) | 290 | 1.302 | [1.116,1.520] | 0.001 |
| LVEF(%) | 290 | 0.975 | [0.956,0.995] | 0.012 |
| Single-pool Kt/V | 290 | 0.813 | [0.490,1.349] | 0.423 |
| Sex |  |  |  |  |
| female | 108 |  |  |  |
| male | 182 | 1.284 | [0.846,1.949] | 0.24 |
| Smoking or Drinking |  |  |  |  |
| no | 219 |  |  |  |
| yes | 71 | 0.953 | [0.598,1.518] | 0.839 |
| Hemodialysis vascular access |  |  |  |  |
| autogenous arteriovenous fistula | 175 |  |  |  |
| long-term cuff catheter | 115 | 1.306 | [0.875,1.948] | 0.191 |
| Dialysis frequency（weekly) |  |  |  |  |
| ＜3 | 54 |  |  |  |
| ≥3 | 236 | 0.741 | [0.461,1.190] | 0.215 |
| Duration of dialysis (h/time) |  |  |  |  |
| 3 | 33 |  |  |  |
| 4 | 257 | 0.486 | [0.284,0.834] | 0.009 |
| Cerebral apoplexy |  |  |  |  |
| no | 250 |  |  |  |
| yes | 40 | 1.678 | [1.028,2.739] | 0.038 |
| Hypertension |  |  |  |  |
| no | 104 |  |  |  |
| grade 1~2 | 32 | 1.952 | [0.998,3.818] | 0.051 |
| grade 3 | 154 | 2.096 | [1.316,3.340] | 0.002 |
| Diabetes |  |  |  |  |
| no | 160 |  |  |  |
| yes | 130 | 3.022 | [2.005,4.554] | ＜0.001 |
| CHD |  |  |  |  |
| no | 250 |  |  |  |
| yes | 40 | 3.577 | [2.344,5.456] | ＜0.001 |
| NYHA |  |  |  |  |
| 0~2 | 129 |  |  |  |
| 3~4 | 161 | 2.197 | [1.421,3.398] | ＜0.001 |
| Metabolic acidosis |  |  |  |  |
| no | 254 |  |  |  |
| yes | 36 | 2.569 | [1.582,4.171] | ＜0.001 |
| Pulmonary artery hypertension |  |  |  |  |
| no | 238 |  |  |  |
| yes | 52 | 1.809 | [1.146,2.854] | 0.011 |
| Serous effusio |  |  |  |  |
| no | 222 |  |  |  |
| yes | 68 | 2.633 | [1.741,3.981] | ＜0.001 |
| History of fracture surgery |  |  | c |  |
| no | 286 |  |  |  |
| yes | 4 | 3.077 | [1.130,8.379] | 0.028 |
| Reasons for entering dialysis |  |  |  |  |
| Diabetes | 109 |  |  |  |
| Nephritis | 67 | 0.471 | [0.276,0.804] | 0.006 |
| Hypertension | 13 | 0.949 | [0.430,2.095] | 0.897 |
| Others(Lupus nephritis、Polycystic kidney.etc) | 101 | 0.264 | [0.160,0.436] | ＜0.001 |
| Antihypertensive drugs, *n*(%) |  |  |  |  |
| no | 100 |  |  |  |
| yes | 190 | 1.185 | [0.784,1.792] | 0.42 |
| Antidiabetic drugs, *n*(%) |  |  |  |  |
| no | 181 |  |  |  |
| yes | 109 | 2.723 | [1.835,4.042] | 0 |
| Calcium tablets or Vitamin D, *n*(%) |  |  |  |  |
| no | 20 |  |  |  |
| yes | 270 | 1.43 | [0.582,3.517] | 0.436 |
| **Note:** *HR, Hazard Ratio; CI, Confidence Interval;* CHD,Coronary-heart-disease; BMI, body mass index; SBP, systolic blood pressure; DBP, Diastolic blood pressure; URR, urea reduction ratio; UFR, ultrafiltration rate; WBC, white blood cell; NEU%, neutrophil ratio; LYM%, lymphocyte ratio; HGB, hemoglobin; CRP, C-reactive protein; A/G, albumin-globulin ratio; Scr, Serum creatinine; Ca, serum calcium P, serum phosphorus; PTH, Parathyroid hormone; TC, Total Cholesterol; PHR, Platelet to high-density lipoprotein cholesterol ratio; LVMI, left atrial diameter; NT-proBNP, N-terminal prohormone of brain natriuretic peptide; LVEF, left ventricular ejection fraction. single-pool Kt/V, spKt/V; K, urea dialytic clearance; t, dialysis time; V, urea distribution volume | | | | |
